# Estimated burden of serious mycoses in Poland

**DOI:** 10.1038/s41598-025-19690-4

**Published:** 2025-10-14

**Authors:** Paweł M. Krzyściak, Magdalena W. Skóra, Agnieszka Gniadek, David W. Denning

**Affiliations:** 1 Department of Infection Control and Mycology, Chair of Microbiology, Faculty of Medicine, Jagiellonian University Medical College, Czysta 18 Street, 31-121 Kraków, Poland; 2 Institute of Nursing and Midwifery, Faculty of Health Sciences, Jagiellonian University Medical College, Kraków, Poland; 3 Manchester Fungal Infection Group, The University of Manchester and Manchester Academic Health Science Centre, Manchester, UK

**Keywords:** Mycoses, Fungal infections, Disease burden, Epidemiology, Candidosis, Aspergillosis, Risk factors, Infection, Epidemiology

## Abstract

Poland, with a population of 38 million, lacks a comprehensive dataset on major fungal infections, leaving the country absent from international fungal burden maps. This study provides a comprehensive analysis of databases of published literature and national demographic and health data to estimate the incidence and prevalence of serious fungal infections in Poland, focusing on the general population and high-risk groups. The estimates indicate a substantial and heterogeneous burden of serious mycoses in Poland. The most common yeast infection is recurrent vulvovaginal candidosis, affecting 12,300–81,600 women annually, while invasive candidosis leads to 280–4600 cases of candidaemia, mostly in intensive care units. Cryptococcosis is very rare, with about 30 cases per year, and *Pneumocystis* pneumonia occurs in 53–959 patients annually. Invasive aspergillosis, with about 5000 estimated cases annually, is the most important mould infection, mainly affecting patients with respiratory disease and immunosuppression. Chronic pulmonary aspergillosis adds about 3,200 cases each year. Mucormycosis is estimated at around 60 cases annually. Other mycoses, such as keratitis and subcutaneous forms, are reported only sporadically. These findings highlight that serious mycoses are a relevant but under-recognized health problem in Poland and underline the need for improved surveillance and targeted public health measures.

**Supplementary Information:**

The online version contains supplementary material available at 10.1038/s41598-025-19690-4.

## Introduction

**Fig. 1:**
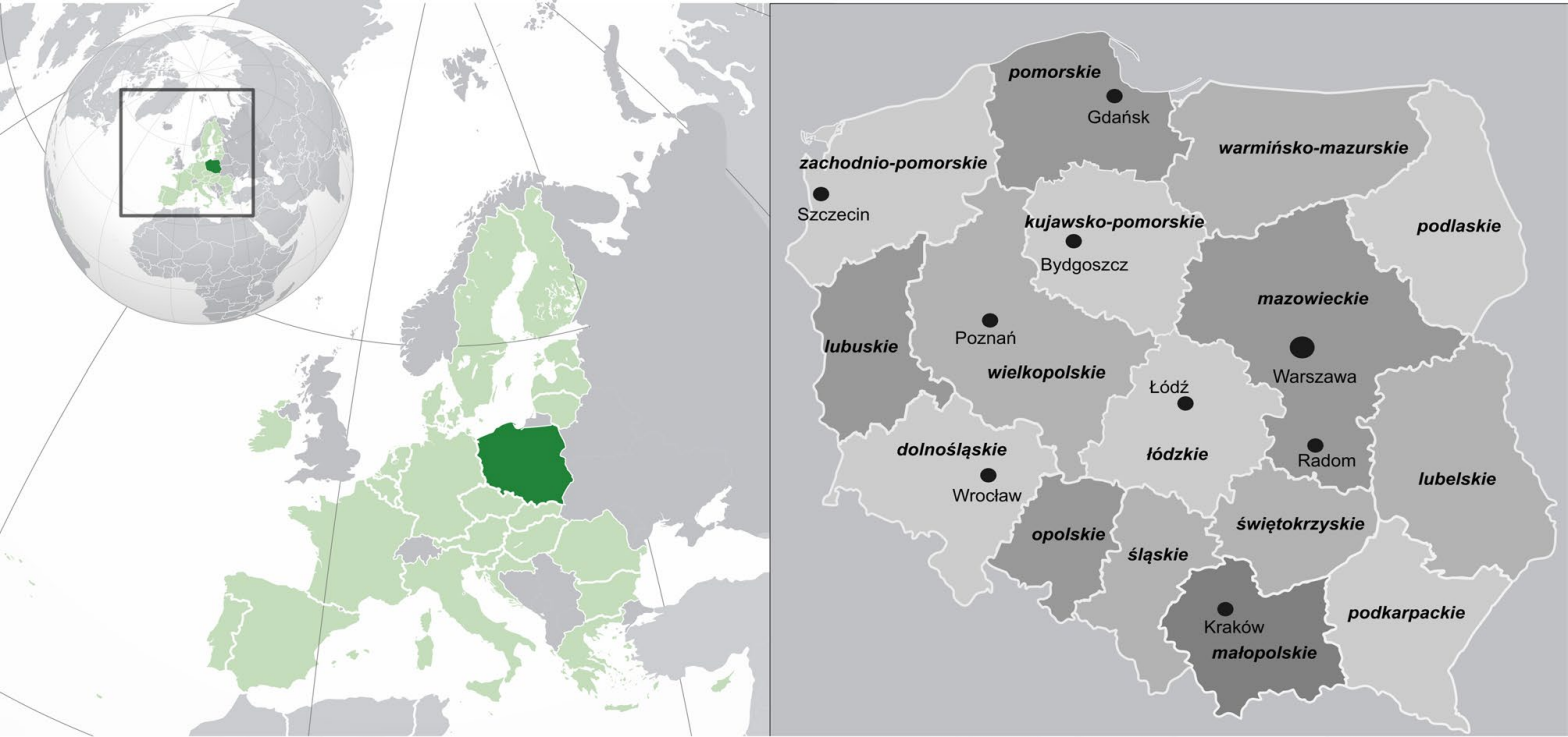
Maps showing Poland (dark green) with voivodeship boundaries and its location within the European Union (light green), including main cities referenced in the text. Composite created in Inkscape (version 1.4.1; https://inkscape.org) using *Voivodeships of Poland.svg* by Odder (Wikimedia Commons; CC BY-SA 4.0; https://commons.wikimedia.org/wiki/File: Voivodeships_of_Poland.svg) and *EU-Poland (orthographic projection).svg* by Rob984 (Wikimedia Commons; CC BY-SA 4.0; https://commons.wikimedia.org/wiki/File: EU-Poland_(orthographic_projection).svg).

Poland, located in Central Europe, spans from the Baltic Sea in the north to the Sudetes and Carpathian mountains in the south (Fig. 1). The country has a population of approximately 38 million and represents an upper-middle income European economy, with a GDP per capita of USD 22,056 in 2023 according to World Bank.

Polish legislation enacted on December 5, 2008, mandates the prevention and control of infectious diseases and requires hospitals to maintain registers of so-called alert pathogens^1^. These include certain fungi, such as fluconazole-resistant *Candida*, *Aspergillus*, and pathogens from blood or cerebrospinal fluid causing invasive infections^2^. Data from these registers are reported to local public health authorities and then aggregated at the national level. While selected statistics are published in annual epidemiological bulletins, there is no publicly accessible, comprehensive national dataset on invasive fungal infections. Consequently, Poland is absent from the “fungal burden” map published by Global Action For Fungal Infections^3^ and Leading International Fungal Education^4^.

## Materials and methods

This study aims to estimate the prevalence of clinically significant fungal infections by analysing scientific publications and demographic data from the region.

### Sources of published data

This study conducted a comprehensive search of both global (PubMed, Scopus) and local (Index Copernicus, Polish Medical Bibliography) databases to identify relevant literature on the incidence and prevalence of serious mycoses in Poland. The search utilized keywords such as “Poland”, “mycoses”, “fungi”, “infection”, and their Polish equivalents. Retrieved references were managed in Zotero and Mendeley. Papers detailing single cases of fungal infections or those that did not allow for the estimation of mycosis incidence (e.g. studies reporting only person-day counts or lacking data on affected populations) were excluded.

Epidemiological and demographic data presented in the following sections were used as background for estimating the frequency of serious mycoses in Poland, by combining population-level and disease-specific information from national and international sources.

### Sources of data on population and its characteristics

General demographic data and information on underlying conditions for mycoses were obtained primarily from publicly available government websites^5^ and bulletins from the National Health Fund (NFZ)^6,7^. Additionally, Poland has a centralized system for monitoring certain infectious diseases, including HIV, tuberculosis (TB), and cancers, which publishes data on disease occurrence^8–11^.

Data analysis employed a dual approach. The first involved global estimates of fungal infection prevalence within exposed populations, while the second relied on morbidity estimates derived from available publications.

### General population data

**Fig. 2:**
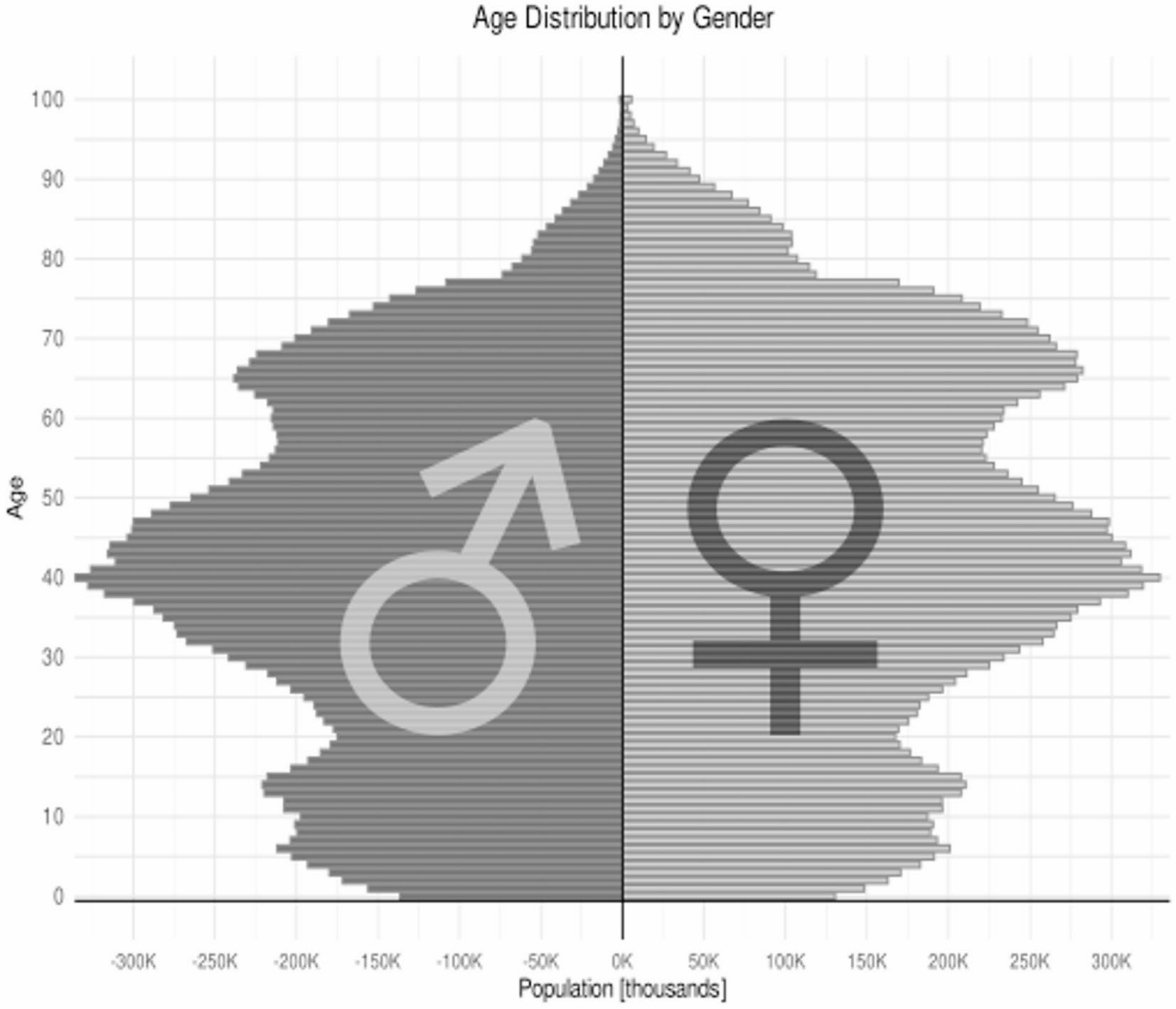
Population structure by demographic characteristics, sex, and age in Poland, 2023 (Based on the Demographic Yearbook)^12^; ♂ - males, ♀ - females.

As of 31 December 2023, Poland’s population was 37,636,508, including 51.7% (19,454,109) females and 19.2% children/adolescents (< 18 years; 7,226,549)^12^. Women aged 15–49 numbered 8,607,733^12^ (Fig. 2). Average life expectancy was 74.7 years for men and 82.0 years for women^13^. The population density was approximately 120/km²^14^.

At the end of 2022, Poland had 896 general hospitals (164,400 beds) and 231 day hospitals (6,500 places for day care)^15^. There were 6,895,900 hospitalizations in general hospitals, including 108,800 admissions to intensive care units (ICUs). Children and adolescents under 18 years of age represented 17.3% (1,191,400 individuals) of hospitalized patients^15^. There were 158,900 licensed physicians and 17,900 laboratory diagnosticians^15^.

### Estimating underlying diseases prevalence linked to mycoses

*Diseases with immunosuppression*. Data from the e-Health Centre indicated that between 2010 and July 2023, 22,043 patients utilized at least two HIV-related healthcare services^16^. As of 31 December 2023, 19,647 (89.1% coverage) individuals were receiving antiretroviral therapy (ART)^17^, with 3,429 AIDS cases (2,396 HIV-positive not on ART; 1,033 on ART with CD4 ≤ 200 cells/µL − 5% of those on ART)^18^. In 2023, 184 new AIDS diagnoses were reported^19^.

Cancer data were obtained from the Maria Skłodowska-Curie National Research Institute of Oncology (KRN)^11,20^and NFZ^21^. In the source reports, prevalence refers to patients alive at year-end, incidence to new diagnoses, and mortality to all deaths regardless of diagnosis year. KRN recorded 181,300 cancer cases and 96,062 deaths in 2022. In 2021, there were 54,552 lung cancers, 3,885 leukaemias (328 in individuals aged 0–19 years), 3,631 non-Hodgkin lymphomas, and 750 Hodgkin’s lymphomas (88 in individuals aged 0–19 years). NFZ data show that 2,012 AML patients received reimbursed chemotherapy in 2022, increasing to 2,195 in 2023, with approximately half also receiving antifungal drugs^21^.

Data on transplant recipients were obtained from “Poltransplant”^22^. In 2023, 977 kidney, 523 liver, 179 heart, and 99 lung transplants were performed in Poland. Hematopoietic stem-cell transplants included 1261 autologous and 790 allogeneic procedures^22^. Additionally, 1,369 corneas were transplanted in 2022^23^.

*Pulmonary diseases.* In 2023, the National Tuberculosis and Lung Diseases Research Institute (IGiChP) reported 4436 TB cases in Poland, 96.1% (4,265) of which were pulmonary tuberculosis (PTB). Additionally, 32 cases were diagnosed among HIV/AIDS patients^24^. The estimated prevalence of sarcoidosis is 6.8 per 100,000 population, with 53% of cases involving the lungs^25^. NFZ data from 2019 recorded 9,963 patients with pulmonary sarcoidosis, including cases with concurrent lymph node involvement^26^. In 2023, asthma affected 2,066,082 people (1,466,626 adults)^27^; difficult-to-treat asthma occurs 17% and severe asthma around 3.7% of patients^28,29^. Monovalent mould sensitization was found in 8.4% of asthma patients, rising to 15.4% in those with severe asthma^30^. In 2019, chronic obstructive pulmonary disease (COPD) was diagnosed in 454,600 people^31^, with 43,900 (9.7%) hospitalised^32^, a proportion within the European range of 6–22%^33^. NFZ data from 2023 reported 2,816 cystic fibrosis (CF) patients, including 1,680 under 18 years of age^34^.

*Other underlying diseases.* In Poland, 3.26 million diabetes cases were reported^35^, with 12.8% requiring hospitalisation^36^. The prevalence of gastro-oesophageal reflux disease (GERD) in Europe ranges from 8.8–25.9%^37^, with similar figures for Poland^38^. In 2019, the number of cases of liver cirrhosis in Poland were estimated at 8,644 (6,080–11,391)^39^.

A summary of the main demographic and underlying disease data is provided in Table 1.

**Table 1:** Population and underlying disease demographics in Poland.

Data			Number	Comments	Refs.
Population			37,636,508	State on 31.12.2023	GUS^12^
Female 15–49 y.o.			19,454,109 8,607,733		
Male			18,182,399		
Children 0–18 y.o.			7,226,549		
Elderly (65 + y.o.)			7,032,590		
Hospitalization			6,895,900	General hospitals in 2022	GUS^15^
ICU			108,800		
Children 0–18 y.o.			1,191,400		
HIV/infection prevalence			22,043 32,935	2023 Total registered cases from 1985^19^	NFZ^16,17^
ART			19,647	89.1% of HIV patients 2023	
AIDS			3429	184 newly diagnosed in 2023^19^	
PTB			4265	2023 3753 new cases; 512 relapses	IGiChP^24^
Bacteriological confirmed Non-confirmed MDR-TB			3,460 805 56	102 with 1 drug R (98 PTB)	
Deaths			461 (452)	2022 mortality rate 1.2 per 100,000 (452 estimation in 2023)	
HIV AIDS			161 32	Based on HIV patients number and Podlasin et al.^48^ IGiChP – TB as a marker disease	
Asthma in adults			1,466,626	2023 (2.07 millions all patients)	NFZ^27^
Difficult-to-treat/uncontrolled Severe asthma			249,326 54,265	17% (GINA) adults 3.7% (GINA) adults (subset of above)	GINA[29] NFZ^27^
Severe asthma therapy			3392	2023, adults	NFZ^27^
COPD patients			454,600	2019 (43,900 hospitalised^32^)	NFZ^31^
Interstitial lung diseases (ILD)			29,238	2019 – ICD10 codes: J84.0; J84.1; J84.8; J84.9; J99.0; J99.1	NFZ^26^
Idiopathic pulmonary Fibrosis			6359	J84.1 ICD-10 code without M05, M33, M34, M35	
ILD with connective tissue disorders			5180	interstitial pneumonia with autoimmune features; rheumatoid arthritis, dermatopolymyositis, systemic sclerosis, primary Sjögren′s syndrome	
Lung cancer			54,552	2021, 14,949 morbidity, 20,729 incidence, and 20,841 deaths^11^	KRN^20^
Haematolymphoid cancers			10,166	2021 (code C81-C96) 2021, 1,033 morbidity, 1,339 incidence, and 1,095 deaths^11^ 2022 (14,456 over 65+)	KRN^135^ NFZ^21^ NFZ^79^
AML CLL			2195 19,839		
Organ transplantation			1806	2023	PT^22^
Renal			977		
Liver			523		
Heart			179		
Lungs			99		
HSC transplantation			2051	2023	
Allogenic HSC			790		
Auto HSC			1,261		

*AML* Acute Myeloid Leukaemia, *ART* Antiretroviral Therapy, *GINA* Global Initiative for Asthma, *GUS* Central Statistical Office of Poland, *HSC* Hematopoietic Stem Cells, *ICU* Intensive Care Unit, *IGiChP* Institute of Tuberculosis and Lung Diseases, *IZWOZ* Institute for Health in Healthcare, *KRN* National Cancer Registry, *MDR-TB* Multi-Drug Resistant Tuberculosis, *NFZ* National Health Fund (Poland), *PT* Poltransplant (Polish Transplantation Registry), *PTB* Pulmonary tuberculosis.

### Estimation of fungal disease burden

Based on the epidemiological and demographic data described in the previous sections, national prevalence or incidence was estimated for each targeted mycosis. Disease-specific rates were obtained from published literature, preferentially from Polish or European studies, and applied to the size of the corresponding at-risk populations in Poland. Where necessary, estimates were adjusted for clinical factors such as treatment status, disease severity, and overlapping risk groups to avoid double counting. In some cases, specific calculation approaches were applied, for example converting lifetime prevalence to annual incidence by dividing by the relevant period of exposure, or correcting for overlaps between conditions such as HIV/AIDS, gastro-oesophageal reflux disease, and liver cirrhosis in oesophageal candidosis estimates. Final burden figures were derived by aggregating estimates across all relevant risk categories. A detailed description of the calculation methodology, including all assumptions and data sources, is available in the Supplementary Data.

For clarity and consistency, the results are presented according to microbiological grouping, starting with yeasts and followed by moulds. Within each group, conditions are ordered by major clinical syndromes. This structure facilitates targeted use of the article as a reference, allowing readers to locate information on a given pathogen category efficiently. In addition, a summary table (Table 2) provides a concise overview of incidence estimates, with averaged ranges to aid readability and facilitate navigation of the Results section.

**Table 2:** Incidence of fungal infections in Poland. Estimation ranges were averaged for clarity and ease of interpretation.

Fungal diseases		Number of fungal infection among patients with underlying diseases					Rate per 100 K	Total (mean)
		HIV/AIDS	Respiratory	Cancer/transplant	Hospitalized ICU	Other		
Vulvovaginal candidosis (VVC)						71,112–294,501	430.2	161,923
Recurrent VVC (rVVC)						12,324–81,577	95.2	35,823
Oesophageal candidosis (EC)		347–1462				4,708–12,695	24.0	9054*
*Candida* fungaemia					280–4590		7.0	2624**
*Candida* peritonitis					*930*	8	2.5	938
Cryptococcosis		5–6			24		0.08	32
*Pneumocystis *pneumonia (PcP)		73–172		328	191 (724)		1.8	669
Invasive aspergillosis (IA)			3193–4339	1509–1665	122–188		14.2	5353
Chronic pulmonary aspergillosis (CPA)			3078		132–196		8.6	3244
Allergic bronchopulmonary aspergillosis (ABPA)			10,337–51,511				82.2	30,924
Severe asthma with fungal sensitization (SAFS)			8357				22.2	8357
Sinus aspergillosis						5312	14.1	5312
Mucormycosis					52–60		0.15	60
Fungal keratitis (FK)						10–22	0.04	16
Total***							**272.1**	**102**,**406**

*Mean including overlapping population at risk; ** mean calculated with averages from different estimation method (details in the text), *** without VVC.

## Results

*Recurrent vulvovaginal candidosis (rVVC).* Vulvovaginal candidosis (VVC) is defined as recurrent when at least four episodes per year or three unrelated to antibiotic use occur^40–42^. In an online survey, Foxman* et al*. found that 29% (France) to 49% (Italy) of women aged 16–65 self-reported a lifetime healthcare diagnosis of VVC^40^. Another approach to estimating VVC is based on the prevalence among pregnant women in Poland (11–23.5%) and the number of births in 2023 (272,000)^43,44^. However, pregnancy is only one of three major independent risk factors for VVC, alongside diabetes and broad-spectrum antibiotic use, which are likely to at least double or triple this estimate. Moreover, a German study based on healthcare records estimated a 5.6% VVC prevalence among women aged 18–60^45^. These different approaches result in a very broad estimated range of VVC cases in Poland, from about 90,000 to 6,000,000 in total, with an annual incidence of 71,000–190,000, or 294,500 when based on the German healthcare data.

To approximate the burden of rVVC, we used the 24.7% recurrence rate found in a Polish study of generally healthy (non-pregnant) women suspected of candidosis^46^ and the proportion reported in Foxman’s survey^40^, where 27.7% of women reported at least one vaginal yeast infection and 24.7% of these had rVVC. These yield estimates of 72,742–81,577 and 17,565–33,283 new rVVC cases annually, respectively, depending on the underlying VVC figures used.

For comparison, Denning *et al*.^42^ estimated the global burden of rVVC using a fixed prevalence of 6% (range 5–8%) among women over 18 years, excluding those post-menopausal on hormone replacement therapy, which for Poland corresponds to 532,400 cases (443,654–709,846) with approximately 15,211 (12,324–19,718) cases annually.

*Oesophageal candidosis (EC).* It is estimated that 20% of HIV/AIDS patients with CD4 counts below 200 × 10⁶/µL who are not receiving ART and 5% of those on ART are susceptible to EC^47^, corresponding to approximately 1462 cases annually. However, a Polish cross-sectional study by Podlasin et al., conducted in the early 2000s and involving 5,156 HIV-positive patients, reported a lower occurrence of fungal infections (mostly EC), although the diagnoses were not all verified and endoscopy was not routinely done^48^. The study estimated a mean prevalence of 0.29% among patients on ART and 1.22% among those not on ART. Other available data suggest that EC affects 14.5–54.7% of AIDS patients in Poland^49,50^. This corresponds to 347–1310 (1462) cases EC among HIV/AIDS patients.

Reflux esophagitis is also associated with EC. Takahashi et al. reported a 1.7% prevalence of EC among patients with upper gastrointestinal disease^51^. A study in northern Poland found that 0.9–2.4% of adults experienced reflux disease symptoms^38^. Among 30 million adults in Poland in 2023, this suggests a potential 4700 to 12,500 cases of reflux-associated EC. European prevalence estimates of GERD (8.8–25.9% population)^37^ would increase these figures ten-fold, resulting in 46,000–135,000 cases. Additionally, Verma et al. estimated that around 2% (0.8–5.8%) of patients with liver cirrhosis develop EC^52^ leading to estimated 69–501 cases of EC. Taking into consideration the overlap between the populations of HIV/AIDS, GERD, and liver cirrhosis patients^53–55^, the estimated number of oesophageal candidosis (EC) cases in Poland is approximately 4,708 to 12,695 cases annually.

*Invasive yeast infection/candidosis.* An older hospital-based study conducted between 1993 and 1998 identified 95 *Candida* spp. strains in the bloodstream of 154,856 patients at a major hospital in the Małopolskie Voivodeship, reflecting an incidence rate of 0.061%^56^. Extrapolating these findings to the national level, with a total of 6,895,900 patients hospitalized in general hospitals^57^, suggests approximately 4,210 cases of *Candida* bloodstream infections nationwide.

Recent multicentre studies reported 302 cases of candidaemia (affecting 294 patients), corresponding to an incidence rate of 0.04 cases per 1,000 patient-days in a hospital setting^58^. Applying this rate in a theoretical estimation model, assuming continuous full occupancy of the 164,400 beds available in Polish general hospitals in 2022 for the maximum of 365 days, yields 60 million patient-days annually and an estimated 2,400 (600–4,200) cases of candidaemia. However, more realistic estimates, derived from two complementary approaches – one based on hospitalized patients in 2022 with an average stay of 5.1 days and the other on a turnover rate of 44.8 patients per bed annually combined with the total number of hospital beds^15^ – suggest a range of 35.2–37.6 million patient-days annually, corresponding to 1,502 *Candida* BSI cases annually, with variability (375–2,629) reflecting incidence rate variability across studies.

Invasive candidosis in adult patients is particularly concerning in ICUs, where early detection of BSIs and sepsis is crucial. Studies conducted in various hospitals in Poland report that the incidence of candidaemia in ICUs ranges from 0.08 to 1.3% (mean 0.9%)^59–62^. Given that 108,800 patients were hospitalized in ICUs in Poland in 2022^15^, this translates to an estimated 979 cases of *Candida* BSI (range: 87–1,414). Using a different approach, combining the 6.9–9.8% occurrence of BSIs among ICU patients^62,63^ with the 7.2% proportion of *Candida* among healthcare-associated bloodstream infections^64^, yields an estimated 541–768 cases of *Candida* BSI annually in Poland. Furthermore, multicentre studies indicate that 30.8% of all *Candida* BSI cases in hospitals originate in ICUs^58^. This proportion suggests that the total incidence of candidaemia in hospitals is approximately three times higher than in ICUs, resulting in an estimated 2,654 cases nationwide (range: 282–4,591).

*Candida peritonitis*. In a three-year study in Szczecin, *Candida* peritonitis occurred in 1 of 89 peritoneal dialysis patients (1.1%)^65^. By the end of 2023, Poland had 20,536 dialysis patients, of whom 766 were on peritoneal dialysis^66^. This suggests an estimated 8 cases of *Candida* peritonitis among peritoneal dialysis patients nationwide. Additionally, a separate study from Szczecin identified 2 cases of candidal peritonitis among 234 ICU patients (0.85%)^60^. Extrapolating this rate, suggests approximately 930 cases of *Candida* peritonitis annualy among ICU patients in Poland.

*Cryptococcosis.* As reported by Jankowska *et al*.^50^ and the ECDC^67^, cryptococcosis, an AIDS marker disease, was found in 1.4–2.2% of patients, corresponding to 3–4 cases among the 184 new AIDS cases in Poland in 2023. Autopsy studies in Warsaw^68,69^ and Wrocław^70^ documented a cryptococcosis prevalence of 8.5–9% among AIDS patients. In 2023, 22 AIDS-related deaths were reported, with two cases likely attributed to cryptococcosis.

Multicentre studies conducted between 2006 and 2007 identified three cases of cryptococcal bloodstream infection, with two caused by *Cryptococcus neoformans* and one by *C*. *laurentii*^58^. Combined with data from transplant recipients and aplastic anaemia patients^71–73^, approximately 24 cases of non-HIV-related cryptococcosis are estimated in Poland.

*Pneumocystosis (PcP).* Studies in young immunocompetent hospitalized children showed that 4.1–13.1% of those with pneumonia symptoms tested positive for *Pneumocystis jirovecii*^74–76^. The interpretation of these findings is uncertain, since a positive result may mean transient carriage or a previous self-limited infection rather than active disease. Nevertheless, it confirms ongoing circulation of *P. jirovecii* in Poland. In contrast, PcP occurred in 2 of 123 immunocompromised children with aplastic anaemia (1.6%)^71^.

Podlasin *et al*. reported PcP rates of 0.19–0.54% among HIV-positive patients on ART and 0.64–1% among those not on ART^48^, translating to 53–130 PcP cases annually. PcP as an AIDS defining diseases occurs in 19.6–35.6%^50,77^ with 36–66 estimated cases annually, which corresponds to the above analysis (slightly increasing the estimation to 73–172 cases).

In a clinical trial cohort of patients with CLL or relapsed NHL, the incidence of PcP was 2.5% among those receiving idelalisib (± co-therapy), compared to 0.2% in those treated only with anti-CD20 antibody or bendamustine-rituximab^78^. Other immunosuppressive regimens used in CLL/NHL, such as Bruton tyrosine kinase inhibitors or intensive chemotherapy, may confer a similar increase in PcP risk. Applying the idelalisib-based incidence rates to the 19,839 CLL cases diagnosed in Poland in 2022^79^, assuming a distribution of 63.3% vs. 36.7% between high-risk and lower-risk regimens, yields an estimated 328 PcP cases in this patient population.

NFZ data on Diagnostic Related Group D18 (atypical pneumonia) recorded only 191 cases of pneumocystosis as a major cause of hospitalizations in 2019, potentially reflecting underreporting due to limitations in ICD-10 coding or diagnostic practices^80^.

Kolbrink *et al*. reported a PcP incidence of 10.5 per 100,000 hospitalizations in Germany, with 27.9% involving HIV-positive patients^81^. Applying this rate to Poland’s 2022 hospitalized population (6,895,900) yields an estimate of approximately 724 PcP cases with approximately 200 expected cases related to HIV, aligning with the calculations above.

*Invasive aspergillosis (IA).* According to Polish morbidity data from 2009 to 2016, collected by PZH, hospitalization rates for invasive pulmonary aspergillosis (IPA) were analysed across all hospitals, excluding psychiatric and military facilities. The dataset included 4,206 hospitalizations of 2,338 patients, with an average of 1.8 hospitalizations per patient. The annual rate of hospitalizations for IPA was estimated at 4.0 per million (95% CI: 3.0–5.0)^82^. Extrapolated to 2023, this corresponds to approximately 151 patients annually, with a range of 112 to 188. Additionally, the NFZ analysis of atypical pneumonia identified 141 patients with pulmonary aspergillosis as the primary cause of hospitalization^80^. However, these figures only reflect primary registrations of aspergillosis, while many additional cases are likely classified under the underlying disease.

Further estimates suggest a substantial number of undiagnosed or misclassified cases in specific at-risk populations. For instance, in patients with lung cancer, the incidence of IPA is estimated at 2.6%^83^, which translates to approximately 1,418 cases annually. Among recipients of allogeneic hematopoietic stem cell transplantation (alloHSCT)^22,71,73,84–87^, the incidence is estimated at 86 cases per year, ranging from 60 to 112. Solid organ transplant recipients contribute an estimated 83 annual cases, with a range of 31 to 135^22,88^. Patients with COPD represent another significant at-risk group, with 1.3 to 3.9% developing IPA^33^. Based on 43,900 hospitalizations for COPD reported in 2023^31^, this corresponds to an estimated 1,141 cases annually, with a range of 571 to 1,712. Among children with cystic fibrosis, pulmonary aspergillosis affects 2.4–2.7% of patients^89^, extrapolating to approximately 40–45 cases annually.

Additionally, in patients with idiopathic pulmonary fibrosis (IPF) and interstitial lung diseases associated with connective tissue disorders, the number of IPA cases in 2019^26,90^ was estimated at 2,582.

Altogether, the estimated annual incidence of IA in Poland is between 4700 and 6000 cases, with a mean of 5350. These findings highlight a significant underdiagnosis of the disease compared to reported figures and underscore the need for improved diagnostic awareness and recording practices to address the true burden of IA in Poland.

*Chronic pulmonary aspergillosis (CPA).* Based on the PZH dataset, the average annual hospitalization rate for non-IPA aspergillosis was 4.4 per million (95% CI: 3.5–5.2)^82^, equivalent to 166 patients in 2023 (range: 132–196). However, CPA is primarily managed in outpatient settings. Among patients with PTB including those often misdiagnosed as “unconfirmed TB,” of whom 19% are estimated to have CPA^91^—the annual incidence of CPA is projected at 1103 cases, with 1003 cases directly attributable to PTB. Furthermore, in the cohort of 9,963 Polish patients diagnosed with pulmonary sarcoidosis^26^, a CPA prevalence of 6%^92^ translates to an additional 598 cases. Assuming all COPD-related hospitalizations represent acute exacerbations—a reasonable assumption given that 9.8% of such cases are associated with CPA^93^—an additional 1377 cases are estimated. This brings the total projected number of CPA cases to 3244.

*Allergic bronchopulmonary aspergillosis (ABPA).* Denning *et al*. assessed the prevalence of ABPA in adults with asthma, finding a prevalence rate of 2.5% (range: 0.7–3.5%)^94^. In Poland, the NFZ reported 1.47 million adults with asthma in 2023, leading to an estimated 36,750 cases of ABPA (range: 10,290–51,450).

European annual reports show that ABPA occurred in 0.7–1.2% of paediatric and 3.1–3.6% of adult cystic fibrosis (CF) patients^95,96^. Similar prevalence rates were confirmed by a CF centre in Warsaw, where 1.1% of paediatric patients (4/374) were diagnosed with ABPA^89^. Based on this, an estimated 12–20 of ABPA in children and 35–41 cases in adults with CF were expected in Poland in 2023.

*Severe asthma with fungal sensitization (SAFS).* A Polish study investigating asthma patients identified monovalent mould sensitization in 8.4% of cases, with rates of 13.1% in the uncontrolled asthma group and 15.4% among those with severe asthma^30^. Based on 2023 NFZ data^27^, and estimation from GINA^29^, this translates to 123,200 asthmatic patients with mould hypersensitivity, and 8,357 SAFS patients in Poland.

*Sinus aspergillosis.* Data from Kraków (1998–2004) indicate that *Aspergillus*-related maxillary sinusitis was found in 2.75% (12/436) of patients diagnosed with chronic rhinosinusitis (CRS)^97^. In Poland, 193,163 individuals were diagnosed with CRS in 2022^99^, which corresponds to an estimated 5,312 cases of sinus aspergillosis annually. However, this figure may be overestimated, because according to the European Position Paper on Rhinosinusitis and Nasal Polyps (EPOS) criteria, when CRS is defined by symptoms confirmed by endoscopy or CT, its prevalence in Europe is 3–6% of the population^98–100^. Applied to the Polish population, this yields 31,050–62,100 CRS cases, corresponding to approximately 854–1,708 cases of sinus aspergillosis.

*Mucormycosis.* The prevalence of mucormycosis-related hospitalizations in Europe ranges from 0.2 cases per million in Denmark to 95 cases per million in Portugal, with an average of 1.6 cases per million (excluding the Portuguese outlier)^101^. Applying this average to Poland suggests that approximately 60 cases should be reported annually. A total number of 21 patients with invasive mucormycosis were reported in Polish hematopoietic cell transplantation and paediatric haemato-oncology centres, including 15 out of 7,788 children (0.19%) and 6 out of 5,237 adults (0.11%)^102^. Based on data from HSCT patients, who, according to Drogari-Apiranthitou *et al*., account for 4.7% of underlying conditions^103^, it is estimated that about 52 cases occurred in 2022, aligning with previous population-based estimates.

*Fungal keratitis (FK).* Keratitis and corneal ulcers accounted for 12.2% of the indications for corneal grafts in Poland^104^. In 2022, a total of 1394 corneal grafts were performed^23^. A single-centre study conducted in Katowice, analyzing samples from adults patients with corneal ulcers (characterized by epithelial defects and stromal inflammation), reported 27.4% had culture-confirmed keratitis. Fungi were identified as the causative agent in 7.1% of cases, though interpolation of data from the last three years in the same study suggests this may reach 15%^105^.

Nowik *et al*. reported that 42.2% of patients with FK underwent corneal grafting^106^. This effectively doubles the estimated number of FK cases to between 8 and 17 annually in Poland. Additionally, the use of confocal microscopy could potentially increase the number of diagnosed cases by 30%. The incidence of FK in Poland is estimated to range between 10 and 22 cases annually, corresponding to 0.03–0.06 cases per 100,000 population. Similarly, the estimated annual incidence of fungal keratitis across Europe remains low, at approximately 0.02 cases per 100,000 population^107^. This European rate would correspond to about 7.5 cases in Poland in 2023.

*Deep skin infection.* Since 1972, four confirmed cases of chromoblastomycosis have been reported in Poland, three of which were confirmed by culture^108^. The incidence of this disease is approximately one case every 10 years. Some Polish mycologists believe that sporotrichosis, once common in the late 19th and early 20th centuries in this region of Europe, has disappeared for unknown reasons^109^. However, a single case of sporotrichosis in a 50-year-old woman, following a mastectomy, has been documented^110^. A literature survey and analysis of mycetoma cases in Europe from 1980 to 2014 revealed no Polish cases^111^. Thus, these conditions remain extremely rare in Poland.

## Discussion

This article focuses on serious fungal infections, excluding dermatophytosis, *Malassezia* superficial infections, and oral candidosis. Although these are the most prevalent conditions, they rarely pose significant clinical challenges and are seldom monitored outside specialized studies. Their occurrence in Poland will be discussed separately.

The burden (incidence and prevalence) of fungal disease in Poland was analysed relying primarily on available literature data. An overall summary of all cases is provided, categorized by annual incidence and prevalence, with rates per 100,000.

Estimating the annual incidence of rVVC is challenging due to limited specific data. Direct calculations, such as those by Denning *et al*.^42^, involve dividing the total number of cases by the time of exposure, assuming a rough even distribution of cases over that period to estimate annual prevalence.

Indirect estimates rely on limited data and the assumption that approximately one-fourth of VVC cases become recurrent^40,46^. Most published estimates of VVC are based on general figures, such as Sobel’s claim that 75% of women experience at least one episode in their lifetime^41^, a broad estimate lacking a defined timeframe and support from population-based cohort studies^112^, making it largely uninformative.

This analysis relies on two primary European data sources. The first is a survey of respondents’ self-reported lifetime VVC cases by Foxman *et al*.^40^, which also required additional calculations by dividing the lifetime period to estimate annual prevalence. The second, a study by Jacob *et al*.^45^, analysed 954,000 practitioner-confirmed cases over 2 years, resulting in higher case numbers. Another European study, based on Swedish medical records, reported a low annual VVC incidence of 3.3–4.6 per 1000 person-years^113^, translating to 29,000–41,000 cases annually in Poland, including 7200–11,400 rVVC cases. These figures likely underestimate the true burden and were therefore omitted, especially as earlier Swedish data indicate a significantly higher VVC occurrence, with at least 80,000 women purchasing antifungal medications in 1999 alone^114^.

The analysis primarily focuses on women of reproductive age, as VVC incidence decreases threefold post-menopause^115^. Nonetheless, postmenopausal cases remain significant and should be included in more precise calculations. The incidence of rVVC in this group, however, is mostly unknown but is likely very low.

The estimated rate of *candidaemia* is 6.6 cases per 100,000 population, which is higher than in some European countries, such as the Netherlands^116^, but lower than in others, such as Italy^117^. Estimates for invasive candidosis (IC) in Poland rely primarily on ICU data from single-centre and multicentre studies. The method of triplicating ICU cases to generalize for all hospitalizations is derived from a multicentre hospital study^58^. *Candida* BSI infections in this study may be overestimated. One single-centre study, using data from the entire hospital, estimated 4,137 nationwide cases BSI annually (11/100,000) which is even higher^118^. Paediatric cases of IC remain relatively low, with rates ranging from 0.8 to 10 per million^119,120^, translating to an estimated 6–72 cases among Poland’s 7.2 million children in 2023. However, the lower estimate is likely an undercount, given that among paediatric cardiac surgery patients, candidaemia incidence ranges from 0.39–0.65%^121–123^, suggesting around 11–18 IC cases annually.

*Cryptococcal disease* is primarily associated with HIV infection, but generally rare in Poland. A longitudinal study conducted in Radom, identified four cases of cryptococcosis among approximately 14,500 post-mortem examinations performed over an 18-year period (1968–1983)^124^. Given that the population of Radom averaged around 180,000 during this period, this translates to an annual incidence of 0.11 cases per 100,000, or approximately 41 cases of cryptococcosis in Poland in 2023. This estimate is in contrast to a two-fold higher number based on prevalence from a European survey^125^, which included non-HIV cases as well.

Polish data on PcP focus primarily on seroprevalence and colonization, rather than clinical cases^126–128^. In immunocompetent adults, *Pneumocystis* colonization rates are 4% in those aged 20–45 and 10.4% in those over 60. In children aged 1–6 years, colonization rates are notably higher at 24.5%. In respiratory disease patients, *Pneumocystis* DNA was detected in 16.2% of cases, often attributed to colonization^128^. Autopsy studies in Warsaw report pneumocystosis frequencies of 11-16.4% among deceased AIDS patients, with 55% of cases diagnosed during life^68,69^. Coinfection, particularly with viruses, should be considered in PcP patients, potentially masking the more serious diagnosis.

*Invasive aspeargillosis*, especially pulmonary, ranges from 8.3 to 15.7% among adult HSCT recipients^84–86^ and from 1.6 to 1.9% among pediatric^71,73^ HSCT recipients, respectively. In 2023, Poland recorded 790 allogeneic HSCT cases. Studies on aspergillosis occurrence in solid organ transplant recipients in Poland are limited. At a Warsaw centre, 3.4% of liver transplant recipients developed invasive aspergillosis (IA)^72^, and among paediatric liver transplant recipients, 3.6% developed IA; however, when liposomal amphotericin B was used as prophylaxis, this rate was only 0.6%^87^. An earlier single-centre study reported that 6.7% of heart transplant patients developed aspergillosis^129^.

Regarding HIV/AIDS patients in Poland, the prevalence of IA varies: Kamiński found that 5.5% of AIDS patients had invasive fungal infections, including 2% with fungal pneumonia, 1.5% with fungal meningitis, and 1.5% with other fungal infections^69^. Leszczyszyn-Pynka identified *Aspergillus* meningitis in 1.3% of AIDS patients (1 out of 76) and in 0.35% of HIV patients (1 out of 284)^49^. Studies of invasive aspergillosis in COPD, acute leukaemia and lung cancer patients have not been published, so these estimates are based on international studies.

The mortality rate among hospitalized aspergillosis cases was remarkably low at approximately 12%^82^, throwing doubt on the accuracy of the diagnosis of IA.

The marked 35-fold difference gap between reported and estimated IA cases in Poland likely reflects the fact that a proportion of cases, even if clinically recognised, are not reported under aspergillosis but rather coded according to the primary underlying disease or as other respiratory conditions. Additional contributors include masking by co-existing diseases, limited availability and use of antigen and PCR (as culture and microscopy are soinsensitive), and patient deaths prior to establishing a diagnosis.

*Mucormycosis* is rare in Europe. The estimation was based solely on data from Greece and the available Polish data in HSCT patients, assuming that in Europe and North America, the most common risk factors for mucormycosis are haematological malignancies and organ transplants, while in Asia, diabetes mellitus is far more prevalent^130^. In Greece, the proportion of mucormycosis risk factors were haematologic malignancy/neutropenia (29.9%), HSCT (4.7%) and other immunodeficiencies (23.4%), diabetes mellitus at 15.9%; immunocompetent patients (after trauma, burns, surgery, accidents) at 22.4%; autoimmune disorders at 7.5%; and other or unknown factors at 8.4%^103^.

*Rare moulds* such as *Scedosporium prolificans* (fungaemia)^131^ and *Fusarium oxysporum* (disseminated infection)^132^ and arthroconidial yeasts *Saprochaete clavata*^133^ have been reported as causative agents of invasive infections in Poland, primarily in haematologic patients. A multicentre study by Nawrot *et al.*, documented one case of *Fusarium incarnatum* infection during the 2006–2007 period^58^. Extrapolating from the patient-day data for *Candida* infections, the estimated number of *Fusarium* cases is approximately nine. Additionally, one case of fusariosis (0.32%) was reported among 308 paediatric HSCT recipients^73^. Given that there were 2,051 HSCT procedures in 2023, with about 12% involving paediatric patients, this would add approximately one case to the national incidence of fusariosis.

The prevalence of *fungal keratitis* is influenced by geographic location, socioeconomic status, occupational risk factors (eye trauma), and contact lens use, with higher incidence rates observed in warmer climates. A retrospective study in Poland (2003–2017) involving 45 patients found eye injuries (15 cases), contact lens use (10 cases), corticosteroid use (6 cases), prior ocular surgeries (7 cases), and ocular surface disorders (7 cases) to be the primary risk factors. Filamentous fungi were identified in 55.6% of cases and yeast in 44.4%^106^.

## Conclusions

 The analysis of the burden of fungal infections underscores significant gaps in Poland’s national fungal infection reporting systems, highlighting the urgent need for improved surveillance and targeted public health strategies. Key strengths of this study include a comprehensive literature review that covers a wide range of fungal infections and at-risk populations, the integration of both global and local data to estimate infection prevalence, and a focus on serious infections that provide valuable insights for public health interventions.

However, the study also reveals notable weaknesses. These include substantial gaps in national data, particularly for at-risk populations, reliance on rough estimates and indirect data due to the absence of comprehensive surveillance, and the potential underestimation of infection rates - especially for recurrent or less-reported conditions. Limited support from population-based cohort studies further complicates efforts to produce accurate estimates, potentially introducing biases. A major challenge pertains to data concerning at-risk populations, such as individuals living with HIV. Most studies on HIV-related fungal infections only provide aggregate data on new cases, without detailed mortality figures, which prevents a comprehensive understanding of morbidity trends within this vulnerable group.

In Poland, the lack of a unified and reliable approach to recording and analysing fungal infection cases remains a critical issue, despite regulations suggesting otherwise. Control bodies, such as the Supreme Audit Office (NIK), emphasize that infection monitoring and reporting systems in controlled hospitals fail to provide complete data. According to NIK, the submitted summary cases may be significantly underestimated^134^. This fragmented data collection hampers effective public health monitoring and limits the capacity for preventive measures and timely interventions.

To gradually improve this situation, priority measures could include: increasing healthcare expenditure for the non-culture-based diagnosis and control of mycoses; revising and modernising reporting systems to ensure completeness and accuracy; adopting standardized case definitions and diagnostic criteria based on international and local guidance; planning and funding national surveillance studies; establishing a central reference centre for fungal diseases—with particular focus on invasive mycoses—to coordinate diagnostics, reference testing, and training; and strengthening laboratory capacity for mycological diagnostics, including molecular methods. Regular training for clinicians and infection control teams should also be implemented to improve detection and reporting practices.

## Supplementary Information

Below is the link to the electronic supplementary material.

Supplementary Material 1

## Data Availability

All public databases on which this article is based are cited and linked in the References section. All data generated or analyzed during this study are included in this published article and its supplementary information files.

## References

[1] Polish Parliament. Act of 5 December 2008 on preventing and combating infections and infectious diseases in humans. *J. Laws Pol.* **234**, 1570 (2008).

[2] Polish Ministry of Health. Obwieszczenie Ministra Zdrowia z dnia 20 Lutego 2024 r. w sprawie ogłoszenia Jednolitego tekstu Rozporządzenia Ministra Zdrowia w sprawie Listy Czynników Slarmowych, Rejestrów Zakażeń Szpitalnych i Czynników Alarmowych Oraz Raportów o Bieżącej Sytuacji epidemiologicznej Szpitala [Notice of the Minister of Health of 20 February 2024 on promulgation of the consolidated text of the Regulation of the Minister of Health on the list of alarm factors, hospital infections registers and alarm factors, and reports on the current epidemiological situation of the hospital]. *J. Laws Pol.* **335** (2024).

[3] GAFFI & Main Page. (2025). https://gaffi.org/

[4] LIFE. Main Page. (2024). http://en.fungaleducation.org

[5] Statistics Poland. Main Page. (2025). https://stat.gov.pl/en/

[6] Centrum e-Zdrowia. *Main Page.* https://ezdrowie.gov.pl/

[7] Narodowy Fundusz Zdrowia. Main Page. (2024). http://www.nfz.gov.pl/

[8] NIZP PZH - PIB. Main Page. (2024). https://www.pzh.gov.pl/

[9] Instytut Gruźlicy i Chorób Płuc. Main Page. (2025). https://www.igichp.edu.pl/

[10] POLTRANSPLANT. Main Page. (2025). https://poltransplant.org.pl/

[11] Krajowy Rejestr Nowotworów. Main Page. http://onkologia.org.pl

[12] Główny Urząd Statystyczny [Statistics Poland]. Baza Demografia. (2024).

[13] Główny Urząd Statystyczny [Statistics Poland], Departament Badań Demograficznych. Life expectancy in Poland—historical tables. Statistics Poland (GUS) (2024).

[14] Główny Urząd Statystyczny [Statistics Poland], Programming, Coordination of Statistical Surveys and Registers Departmen. Area and population in the territorial profile in 2023. (2023). https://stat.gov.pl/download/gfx/portalinformacyjny/pl/defaultaktualnosci/5468/7/20/1/powierzchnia_i_ludnosc_w_przekroju_terytorialnym_w_2023_roku.pdf

[15] Główny Urząd Statystyczny [Statistics Poland], Social Surveys and Labour Market Department. Health and health care in 2022. (2023). https://stat.gov.pl/download/gfx/portalinformacyjny/pl/defaultaktualnosci/5513/1/13/1/zdrowie_i_ochrona_zdrowia_2022_2.pdf

[16] Centrum e-Zdrowia. Podstawowe dane dotyczące pacjentów z AIDS [Basic data on patients with AIDS]. (2023).

[17] Minister of Digital Affairs. Liczba osób leczonych antyretrowirusowo w poszczególnych klinikach na koniec 2023 r. [Number of people receiving antiretroviral treatment in individual clinics as of the end of 2023]. (2024). https://dane.gov.pl/pl/dataset/3053,leczenie-antyretrowirusowe-osob-zyjacych-z-wirusem/resource/55722/table?page=1&per_page=20&q=&sort=.

[18] Parczewski, M. et al. Meeting the WHO 90% target: antiretroviral treatment efficacy in Poland is associated with baseline clinical patient characteristics. *J. Int. AIDS Soc.* **20**, 21847 (2017). 28715160 10.7448/IAS.20.1.21847 PMC5577695

[19] Niedźwiedzka-Stadnik, M., Nowakowska-Radziwonka, E. & Zakażenia HIV i zachorowania na AIDS w Polsce w 2023 roku [HIV Infections and AIDS Morbidity in Poland in 2023]. (2024). https://wwwold.pzh.gov.pl/oldpage/epimeld/hiv_aids/index.htm 10.32394/pe/197855 40101012

[20] Didkowska, J., Barańska, K., Miklewska, M. J. & Wojciechowska, U. Cancer incidence and mortality in Poland in 2023. *Nowotw J. Oncol.* **74**, 75–93 (2024).

[21] Centrum e-Zdrowia. Chemioterapia dla pacjentów z rozpoznaniem ICD-10: C92.0, C67 i C61 [Chemotherapy for Patients Diagnosed with ICD-10 Codes C92.0, C67, and C61]. (2024).

[22] Poltransplant Information Bulletin. (2024). https://files.poltransplant.org.pl/Biuletyn_2024_www.pdf?utm_source=Poltransplant&utm_medium=biuletyn&utm_campaign=Biuletyn+Informacyjny+Poltransplantu+2024

[23] Poltransplant Information Bulletin. https://bit.ly/Biuletyn2023. (2023).

[24] Korzeniewska-Koseła, M. Tuberculosis and lung diseases in Poland in (2023). https://www.igichp.edu.pl/wp-content/uploads/2024/08/Biuletyn_2024.pdf (2024).

[25] Bogdan, M. et al. Hospitalizations of sarcoidosis patients before and during the COVID-19 pandemic in Poland. *Pol. Arch. Intern. Med.*10.20452/pamw.16618 (2024). 38164521 10.20452/pamw.16618

[26] Centrum e-Zdrowia. Epidemiologia choroby śródmiąższowej płuc [Epidemiology of Interstitial Lung Disease]. (2021).

[27] Narodowy Fundusz Zdrowia. NFZ o zdrowiu. Astma. [NFZ on Health. Asthma]. (2024). https://ezdrowie.gov.pl/portal/home/badania-i-dane/zdrowe-dane/raporty/nfz-o-zdrowiu-astma

[28] Hekking, P. P. W. et al. The prevalence of severe refractory asthma. *J. Allergy Clin. Immunol.* **135**, 896–902 (2015). 25441637 10.1016/j.jaci.2014.08.042

[29] Global Initiative for Asthma. Global Strategy for Asthma Management and Prevention. (2024). https://ginasthma.org/2024-report/

[30] Kołodziejczyk, K., Bożek, A., Jarząb, J. & Gawlik, R. The clinical differences of asthma in patients with molds allergy. *Adv. Respir Med.* **84**, 81–86 (2016). 10.5603/PiAP.2016.0005 27238165

[31] Narodowy Fundusz Zdrowia. NFZ o zdrowiu. Choroby odtytoniowe. [NFZ on Health. Tobacco-related Diseases.]. (2021). https://ezdrowie.gov.pl/portal/home/badania-i-dane/zdrowe-dane/raporty/nfz-o-zdrowiu-choroby-odtytoniowe

[32] Jankowski, M. et al. Epidemiological characteristics of 101,471 patients hospitalized with chronic obstructive pulmonary disease (COPD) in Poland in 2019: multimorbidity, duration of hospitalization, in-hospital mortality. *Adv. Respir Med.* **91**, 368–382 (2023). 37736975 10.3390/arm91050029 PMC10514800

[33] Hammond, E. E., McDonald, C. S., Vestbo, J. & Denning, D. W. The global impact of *Aspergillus* infection on COPD. *BMC Pulm Med.* **20**, 241 (2020). 32912168 10.1186/s12890-020-01259-8 PMC7488557

[34] Centrum e-Zdrowia. Liczba pacjentów z rozpoznaniem mukowiscydozy. [Number of patients diagnosed with cystic fibrosis]. (2024).

[35] Centrum e-Zdrowia. Świadczenia związane z cukrzycą [Services related to diabetes]. (2024).

[36] Narodowy Fundusz Zdrowia. NFZ o zdrowiu. Cukrzyca. [NFZ on Health. Diabetes]. (2019). https://ezdrowie.gov.pl/pobierz/nfz_o_zdrowiu_cukrzyca

[37] El-Serag, H. B., Sweet, S., Winchester, C. C. & Dent, J. Update on the epidemiology of gastro-oesophageal reflux disease: A systematic review. *Gut* **63**, 871–880 (2014). 23853213 10.1136/gutjnl-2012-304269 PMC4046948

[38] Dowgiałło-Wnukiewicz, N., Frask, A., Lech, P. & Michalik, M. Study of the prevalence of gastroesophageal reflux symptoms and the role of each in relation to the GERD impact scale, based on a population of patients admitted for laparoscopic surgery compared to a control group. *Videosurg. Miniinvas. Tech.* **13**, 199–211 (2018). 10.5114/wiitm.2018.75909 PMC6041586 30002752

[39] Wang, Y. et al. Global burden of liver cirrhosis 1990–2019 and 20 years forecast: Results from the global burden of disease study 2019. *Ann. Med.* **56**, 2328521 (2024). 38727511 10.1080/07853890.2024.2328521 PMC11089929

[40] Foxman, B., Muraglia, R., Dietz, J. P., Sobel, J. D. & Wagner, J. Prevalence of recurrent vulvovaginal candidiasis in 5 European countries and the United States: Results from an internet panel survey. *J. Low Genit. Tract. Dis.* **17**, 340–345 (2013). 23486072 10.1097/LGT.0b013e318273e8cf

[41] Sobel, J. D. et al. Vulvovaginal candidiasis: Epidemiologic, diagnostic, and therapeutic considerations. *Am. J. Obstet. Gynecol.* **178**, 203–211 (1998). 9500475 10.1016/s0002-9378(98)80001-x

[42] Denning, D. W., Kneale, M., Sobel, J. D. & Rautemaa-Richardson, R. Global burden of recurrent vulvovaginal candidiasis: A systematic review. *Lancet Infect. Dis.* **18**, e339–e347 (2018). 30078662 10.1016/S1473-3099(18)30103-8

[43] Mikołajczyk, K., Zimmer, M., Tomiałowicz, M. & Fuchs, T. Ocena częstości wystopowania grzybicy pochwy u ciężarnych na terenie Dolnego Śla̧ska [Frequency of vulvovaginal candidiasis in pregnant women in area of Lower Silesia]. *Mikol Lek*. **13**, 175–179 (2006).

[44] Machalski, T., Der, J. & Sikora, J. Kandydoza Pochwy u ciężarnych. [Vaginal candidiasis in pregnant women]. *Mikol Lek*. **13**, 185–186 (2006).

[45] Jacob, L., John, M., Kalder, M. & Kostev, K. Prevalence of vulvovaginal candidiasis in gynecological practices in germany: A retrospective study of 954,186 patients. *Curr Med. Mycol* **4**, 6–11 (2018). 10.18502/cmm.4.1.27 PMC6101156 30186987

[46] Nawrot, U., Grzybek-Hryncewicz, K., Zielska, U., Czarny, A. & Podwińska, J. The study of cell-mediated immune response in recurrent vulvovaginal candidiasis. *FEMS Immunol. Med. Microbiol.* **29**, 89–94 (2000). 11024346 10.1111/j.1574-695X.2000.tb01509.x

[47] Buchacz, K. et al. AIDS-defining opportunistic illnesses in US patients, 1994–2007: A cohort study. *AIDS Lond. Engl.* **24**, 1549–1559 (2010). 10.1097/QAD.0b013e32833a3967 20502317

[48] Podlasin, R. B. et al. Opportunistic infections and other AIDS-defining illnesses in Poland in 2000–2002. *Infection* **34**, 196–200 (2006). 16896577 10.1007/s15010-006-5030-y

[49] Leszczyszyn-Pynka, M. Profil kliniczny zakażeń drobnoustrojami oportunistycznymi u chorych zakażonych HIV–obserwacje własne. [Clinical profile of infections caused by opportunistic microorganisms in HIV-positive patients–own observation]. *Przegląd Epidemiol.* **55** (Suppl 3), 109–116 (2001). 11984936

[50] Jankowska, M., Lemańska, M., Trocha, H., Gesing, M. & Smiatacz, T. Infekcje oportunistyczne występujące u pacjentów zakażonych HIV Hospitalizowanych w Klinice Chorób Zakaźnych AMG. [Opportunistic infections in HIV-positive patients hospitalized in the Clinic of Infectious Diseases AMG]. *Przegląd Epidemiol.* **55** (Suppl 3), 125–128 (2001). 11984938

[51] Takahashi, Y. et al. Long-Term trends in esophageal candidiasis prevalence and associated risk factors with or without HIV infection: Lessons from an endoscopic study of 80,219 patients. *PLoS ONE*. **10**, e0133589 (2015). 26208220 10.1371/journal.pone.0133589 PMC4514810

[52] Verma, N. et al. Prevalence, predictors, and outcomes of esophageal candidiasis in cirrhosis: An observational study with systematic review and meta-analysis (CANDID-VIEW). *J. Clin. Exp. Hepatol.* **12**, 118–128 (2022). 35068792 10.1016/j.jceh.2021.03.005 PMC8766531

[53] Bader, M. & Yi, Y. Gastroesophageal reflux disease in HIV-Infected adults: Prevalence and risk factors of moderate-severe or frequent symptoms. *Open. Forum Infect. Dis.* **3**, 1529 (2016).

[54] Zhang, J. et al. Gastroesophageal reflux in cirrhotic patients without esophageal varices. *World J. Gastroenterol. WJG*. **17**, 1753–1758 (2011). 21483637 10.3748/wjg.v17.i13.1753 PMC3072641

[55] Leszczyszyn-Pynka, M. et al. Hepatitis C coinfection adversely affects the life expectancy of people living with HIV in Northwestern Poland. *Arch. Med. Sci.* **14**, 554–559 (2018). 29765442 10.5114/aoms.2016.58744 PMC5949897

[56] Kedzierska, J., Szygula, M. & Dolezal, M. [Proportion of fungi among isolated microorganisms from blood of patients treated in the teaching departments of the university hospital in Cracow in 1993–1998]. *Med. Dosw. Mikrobiol*. **52**, 197–205 (2000). 11107793

[57] Główny Urząd Statystyczny. [Statistics Poland] Wydział Statystyki Zdrowia, Ośrodek Statystyki Zdrowia i Ochrony Zdrowia. Health and Health Care in 2018. https://stat.gov.pl/en/topics/health/health/health-and-health-care-in-2018, (2019). 1,9.html.

[58] Nawrot, U. et al. Candidaemia in Polish hospitals—a multicentre survey. *Mycoses* **56**, 576–581 (2013). 23565662 10.1111/myc.12077

[59] Kolpa, M., Walaszek, M., Gniadek, A., Wolak, Z. & Dobroś, W. Incidence, microbiological profile and risk factors of healthcare-associated infections in intensive care units: A 10 year observation in a provincial hospital in Southern Poland. *Int. J. Environ. Res. Public. Health*. **15**, 112 (2018). 29324651 10.3390/ijerph15010112 PMC5800211

[60] Wieder-Huszla, U. Monitorowanie zakażeń szpitalnych na oddziale intensywnej terapii medycznej [Monitoring of hospital infections in the medical intensive care unit]. *Rocz Pomor Akad. Med. W Szczecinie*. **56**, 20–29 (2010). 22053623

[61] Wałaszek, M., Różańska, A., Bulanda, M. & Wójkowska-Mach, J. Alarming results of nosocomial infections in a multicenter program of surveillance in Polish intensive care units. *Przegląd Epidemiol.* **72**, 33–44 (2018). 29667378

[62] Rafa, E., Wałaszek, M. Z., Wałaszek, M. J., Domański, A. & Różańska, A. The incidence of healthcare-associated infections, their clinical forms, and microbiological agents in intensive care units in Southern Poland in a multicentre study from 2016 to 2019. *Int. J. Environ. Res. Public. Health*. **18**, 2238 (2021). 33668288 10.3390/ijerph18052238 PMC7956275

[63] Tomaszewski, D., Rybicki, Z. & Duszyńska, W. The Polish prevalence of infection in intensive care (PPIC): A one-day point prevalence multicenter study. *Adv. Clin. Exp. Med.* **28**, 907–912 (2019). 30986000 10.17219/acem/94147

[64] European Centre for Disease Prevention and Control. *Point Prevalence Survey of Healthcare-Associated Infections and Antimicrobial Use in European Acute Care Hospitals, 2022–2023* (Publications Office, 2024).

[65] Kabat-Koperska, J., Gołembiewska, E. & Ciechanowski, K. Peritoneal dialysis-related peritonitis in the years 2005–2007 among patients of the Peritoneal Dialysis Clinic of the Department of Nephrology, Transplantology and Internal Medicine, Pomeranian Medical University in Szczecin. *Pol. Arch. Med. Wewnętrznej*. **118**, 694–698 (2005). 19202946

[66] Dębska-Ślizień, A. et al. Aktualny stan leczenia nerkozastępczego w Polsce—2023. *Pol. Nephrol. Dial.* **28**, 3–18 (2024).

[67] WHO Regional Office for Europe, European Centre for Disease Prevention and Control. HIV/AIDS Surveillance in Europe 2022–2021 Data. (2022). https://www.ecdc.europa.eu/en/publications-data/hiv-aids-joint-report-surveillance-2021-data

[68] Walewska-Zielecka, B. Korelacje rozpoznań klinicznych i morfologicznych w 55 przypadkach zgonów w przebiegu AIDS. [Correlation of clinical and morphological diagnoses in 55 fatal AIDS cases]. *Przegląd Epidemiol.* **49**, 353–359 (1995). 8868192

[69] Kamiński, Z. Various opportunistic infections and neoplasms in patients dying of AIDS in the last 12 years–report based on pathomorphological investigations. *Med. Sci. Monit.* **7**, 421–426 (2001). 11386019

[70] Janocha-Litwin, J., Zińczuk, A., Serafińska, S., Szymanek-Pasternak, A. & Simon, K. Analysis of deaths among HIV-Infected patients hospitalized in 2009–2018 in main centre of infectious disease in region of lower Silesia in Poland, detailing lesions in the central nervous system. *Med. (Mex)*. **58**, 270 (2022). 10.3390/medicina58020270 PMC8875164 35208594

[71] Pawelec, K., Salamonowicz, M., Panasiuk, A., Matysiak, M. & Demkow, U. Respiratory and systemic infections in children with severe aplastic anemia on immunosuppressive therapy. *Adv. Exp. Med. Biol*. **788**, 417–425 (2013). 10.1007/978-94-007-6627-3_57 23836007

[72] Pacholczyk, M. J., Lagiewska, B., Lisik, W., Wasiak, D. & Chmura, A. Invasive fungal infections following liver transplantation—risk factors, incidence and outcome. *Ann. Transpl.* **16**, 14–16 (2011). 10.12659/aot.881989 21959504

[73] Styczyński, J. et al. Inwazyjne zakażenia grzybicze u dzieci i młodzieży po przeszczepieniu komórek krwiotwórczych. [Invasive fungal infections in children and adolescents after hematopoietic stem cell transplantation]. *Postępy Nauk. Med.* **28**, 361–366 (2015).

[74] Gowin, E. et al. Assessment of the usefulness of multiplex real-time PCR tests in the diagnostic and therapeutic process of pneumonia in hospitalized Children: A single-center experience. *BioMed. Res. Int.* 1–8. (2017). 10.1155/2017/8037963 PMC5274672 28182108

[75] Gołąb, E., Rozej-Bielicka, W. & Pancer, K. Evaluation of the frequency of *Pneumocystis**jirovecii* occurrence in a group of children hospitalized for acute respiratory infections. *Wiad Parazytol*. **57**, 93–96 (2011). 21682093

[76] Zając-Spychała, O. et al. *Pneumocystis* pneumonia in children—the relevance of chemoprophylaxis in different groups of immunocompromised and immunocompetent paediatric patients. *Cent. Eur. J. Immunol.* **40**, 91–95 (2015). 26155189 10.5114/ceji.2015.50839 PMC4472545

[77] Szmulik-Misiurek, K., Niedźwiedzka-Stadnik, M. & Rosińska, M. HIV and AIDS in Poland in 2018. *Przegl Epidemiol.* **74**, 223–238 (2020). 33112106 10.32394/pe.74.18

[78] Sehn, L. H. et al. A retrospective analysis of *Pneumocystis**jirovecii* pneumonia infection in patients receiving idelalisib in clinical trials. *Blood* **128**, 3705–3705 (2016).

[79] Centrum e-Zdrowia. Świadczenia z rozpoznaniem przewlekłej białaczki limfocytowej. [Services for patients with chronic lymphocytic leukemia]. (2023). https://ezdrowie.gov.pl/23413

[80] Centrum e-Zdrowia. Informacje o zapaleniu płuc. Dane za rok 2019. [Information on pneumonia—data for 2019]. (2021). https://ezdrowie.gov.pl/8048

[81] Kolbrink, B. et al. Evolving epidemiology of *Pneumocystis* pneumonia: Findings from a longitudinal population-based study and a retrospective multi-center study in Germany. *Lancet Reg. Health Eur.* **18**, 100400 (2022). 35814339 10.1016/j.lanepe.2022.100400 PMC9257643

[82] Tarka, P. et al. Epidemiology of pulmonary aspergillosis in hospitalized patients in Poland during 2009–2016. In *Advances in Pulmonary Medicine: Research and Innovations* (ed Pokorski, M.) 73–80 (Springer, Cham, doi:10.1007/5584_2019_347. (2019). 10.1007/5584_2019_347 30919263

[83] Yan, X. et al. Clinical characteristics of 45 patients with invasive pulmonary aspergillosis. *Cancer* **115**, 5018–5025 (2009). 19637340 10.1002/cncr.24559

[84] Karakulska-Prystopiuk, E., Dwilewicz-Trojaczek, J., Król, M. & Jędrzejczak, W. Ocena występowania zakażeń u chorych po allo-HCT. [Evaluation of infections in patients after allo-HCT]. In Proceedings of the I Polish Conference on Hematopoietic Cell Transplantation, Warsaw, Poland, 7-9 May 2015 p. 37 Polish Society of Hematology (2015).

[85] Nawrot, U., Ussowicz, M., Kałwak, K., Chybicka, A. & Pajączkowska, M. Ocena przydatności galaktomannanu *Aspergillus* w diagnostyce inwazyjnej aspergilozy u pacjentów po przeszczepie komórek hematopoetycznych. [Aspergillus galactomannan antigen testing in detection of invasive aspergillosis in hematopoietic stem cell transplant recipients]. *Mikol Lek*. **16**, 73–76 (2009).

[86] Gil, L. et al. Increased risk for invasive aspergillosis in patients with lymphoproliferative diseases after autologous hematopoietic SCT. *Bone Marrow Transpl.* **43**, 121–126 (2009). 10.1038/bmt.2008.303 18794866

[87] Teisseyre, J. et al. Aspergillosis in children after liver transplantation: Single center experience. *Pediatr. Transpl.* **11**, 868–875 (2007). 10.1111/j.1399-3046.2007.00754.x 17976121

[88] Neofytos, D. et al. Invasive aspergillosis in solid organ transplant patients: Diagnosis, prophylaxis, treatment, and assessment of response. *BMC Infect. Dis.* **21**, 296 (2021). 33761875 10.1186/s12879-021-05958-3 PMC7989085

[89] Walicka-Serzysko, K. & Sands, D. The clinical presentation of pulmonary aspergillosis in children with cystic fibrosis—preliminary report. *Dev. Period Med.* **19**, 66–79 (2015). 26003072

[90] Liu, Y. et al. Clinical features and risk factors of invasive pulmonary aspergillosis in interstitial lung disease patients. *BMC Pulm Med.* **24**, 602 (2024). 39633326 10.1186/s12890-024-03430-x PMC11619705

[91] Denning, D. W., Cole, D. C. & Ray, A. New Estimation of the prevalence of chronic pulmonary aspergillosis (CPA) related to pulmonary TB—a revised burden for India. *IJID Reg.* **6**, 7–14 (2023). 36568568 10.1016/j.ijregi.2022.11.005 PMC9772841

[92] Denning, D. W., Pleuvry, A. & Cole, D. C. Global burden of chronic pulmonary aspergillosis complicating sarcoidosis. *Eur. Respir J.* **41**, 621–626 (2013). 22743676 10.1183/09031936.00226911

[93] Palanivel, J. et al. Prevalence and risk factors for chronic pulmonary aspergillosis in chronic obstructive pulmonary disease patients with acute exacerbations. *Monaldi Arch. Chest Dis.* **95**, 2927 (2024). 38517152 10.4081/monaldi.2024.2927

[94] Denning, D. W., Pleuvry, A. & Cole, D. C. Global burden of allergic bronchopulmonary aspergillosis with asthma and its complication chronic pulmonary aspergillosis in adults. *Med. Mycol.* **51**, 361–370 (2013). 23210682 10.3109/13693786.2012.738312

[95] Zolin, A. et al. & ECFSPR Annual Report. (2021). https://www.ecfs.eu/sites/default/files/Annual%20Report_2021_09Jun2023_ECFSPR_final.pdf (2023).

[96] Zolin, A. et al. & ECFSPR Annual Report. (2022). https://www.ecfs.eu/sites/default/files/Annual%20Report_2022_ECFSPR_20240603.pdf (2024).

[97] Zapała, J., Szuta, M., Dzierwa-Gawron, A., Budak, A. & Zarzecka, J. Aspergiloza zatok przynosowych u chorych leczonych w Klinice Chirurgii Szczękowo-Twarzowej IS CM UJ w Krakowie. [Aspergillosis of paranasal sinuses in patients treated in the Department of Maxillofacial Surgery of the Jagiellonian University in Cracow]. *Mikol Lek*. **13**, 19–23 (2006).

[98] Hastan, D. et al. Chronic rhinosinusitis in Europe–an underestimated disease. A GA^2^ LEN study. *Allergy* **66**, 1216–1223 (2011). 21605125 10.1111/j.1398-9995.2011.02646.x

[99] Fokkens, W. J. et al. European position paper on rhinosinusitis and nasal polyps 2020. *Rhinol. J.* **58**, 1–464 (2020). 10.4193/Rhin20.600 32077450

[100] Bakhshaee, M., Fereidouni, M., Nourollahian, M. & Movahed, R. The presence of fungal-specific IgE in serum and sinonasal tissue among patients with sinonasal polyposis. *Eur. Arch. Oto-Rhino-Laryngol. Off J. Eur. Fed. Oto-Rhino-Laryngol. Soc. EUFOS Affil Ger. Soc. Oto-Rhino-Laryngol - Head Neck Surg.* **271**, 2871–2875 (2014). 10.1007/s00405-014-2882-0 24510177

[101] Prakash, H. & Chakrabarti, A. Global epidemiology of mucormycosis. *J. Fungi*. **5**, 26 (2019). 10.3390/jof5010026 PMC6462913 30901907

[102] Styczyński, J. et al. Clinical spectrum and outcome of invasive mucormycosis in children and adults: Polish experience of the decade 2010–2019. *Acta Haematol. Pol.* **51**, 157–163 (2020).

[103] Drogari-Apiranthitou, M. et al. Epidemiology of mucormycosis in Greece; Results from a nationwide prospective survey and published case reports. *J. Fungi*. **9**, 425 (2023). 10.3390/jof9040425 PMC10142618 37108880

[104] Kozioł, M., Nowak, M. S., Udziela, M. & Szaflik, J. P. The association between diabetes mellitus and keratoplasty in Poland in the years 2013–2017. *Int. J. Environ. Res. Public. Health*. **18**, 9767 (2021). 34574703 10.3390/ijerph18189767 PMC8470624

[105] Ulfik, K. et al. Seven-year analysis of microbial keratitis tendency at an ophthalmology department in Poland: A single-center study. *J. Ophthalmol.* **2020**, 8851570 (2020). 10.1155/2020/8851570 PMC7803137 33489345

[106] Nowik, K. E., Wylęgała, A., Nowik, K. & Wylęgała, E. A single-centre retrospective observational study of fungal keratitis in Poland with a review of findings in Europe. *Ann. Agric. Environ. Med.* **27**, 343–347 (2020). 32955212 10.26444/aaem/109414

[107] Ong, H. S. et al. Altered patterns of fungal keratitis at a London ophthalmic referral hospital: An eight-year retrospective observational study. *Am. J. Ophthalmol.* **168**, 227–236 (2016). 27287820 10.1016/j.ajo.2016.05.021 PMC4977014

[108] Pindycka-Piaszczyńska, M. et al. Chromoblastomycosis as an endemic disease in temperate europe: First confirmed case and review of the literature. *Eur. J. Clin. Microbiol. Infect. Dis.* **33**, 391–398 (2014). 24048727 10.1007/s10096-013-1969-7

[109] Białynicki-Birula, R., Szymczak, T., Baran, E. & Kołodziej, T. Clinical features of lymphocutaneous sporotrichosis from the beginning of the 20th century preserved in moulages. *Mikol Lek*. **10**, 187–191 (2003).

[110] Wroblewska, M. et al. Infection by a dimorphic fungus *Sporothrix schenckii* in an immunocompromised patient. *Infection* **33**, 289–291 (2005). 16091902 10.1007/s15010-005-4123-3

[111] Buonfrate, D. et al. Autochthonous cases of mycetoma in Europe: Report of two cases and review of literature. *PLoS ONE*. **9**, e100590 (2014). 24963778 10.1371/journal.pone.0100590 PMC4070928

[112] Rathod, S. D. & Buffler, P. A. Highly-cited estimates of the cumulative incidence and recurrence of vulvovaginal candidiasis are inadequately documented. *BMC Womens Health*. **14**, 43 (2014). 24612727 10.1186/1472-6874-14-43 PMC3975582

[113] Jansåker, F., Frimodt-Møller, N., Li, X. & Sundquist, K. Novel risk factors associated with common vaginal infections: A nationwide primary health care cohort study. *Int. J. Infect. Dis.* **116**, 380–386 (2022). 35038603 10.1016/j.ijid.2022.01.021

[114] Mårdh, P. A., Wågström, J., Landgren, M. & Holmén, J. Usage of antifungal drugs for therapy of genital *Candida* infections, purchased as Over-the‐Counter products or by prescription: 1. Analyses of a unique database. *Infect. Dis. Obstet. Gynecol.* **12**, 91–97 (2004). 15739823 10.1080/10647440400003873 PMC1784597

[115] Durda-Masny, M. et al. Trends over time in age at sexual debut among Polish women and underlying socio-economic determinants. *Anthropol. Anz*. 185–191. 10.1127/ANTHRANZ/2018/0853 (2018). 10.1127/anthranz/2018/0853 29892777

[116] Buil, J. B., Meijer, E. F. J., Denning, D. W., Verweij, P. E. & Meis, J. F. Burden of serious fungal infections in the Netherlands. *Mycoses* **63**, 625–631 (2020). 32297377 10.1111/myc.13089 PMC7318641

[117] Bassetti, M. et al. Estimated burden of fungal infections in Italy. *J. Infect.* **76**, 103–106 (2018). 28780007 10.1016/j.jinf.2017.07.008

[118] Kędzierska, J., Węgrzyn, J., Kędzierska, A. & Pietrzyk, A. Bakteryjne i grzybicze zakażenia krwi u chorych w oddziałach klinicznych Szpitala Uniwersyteckiego w Krakowie. [Bacterial and fungal bloodstream infections in patients hospitalized in clinical wards of the University Hospital in Kraków]. *Med. Dośw Mikrobiol*. **55**, 259–270 (2003). 14702668

[119] Małafiej, E., Adamiec, A. C. & Tworzyańska, U. Microbial profile and drug resistance of* Candida *strains isolated from the blood of children: An 11-year study. *Mycoses* **52**, 149–153 (2009). 18627468 10.1111/j.1439-0507.2008.01560.x

[120] Dzierzanowska-Fangrat, K. et al. Candidaemia in a Polish tertiary paediatric hospital, 2000 to 2010. *Mycoses* **57**, 105–109 (2014). 23834472 10.1111/myc.12107

[121] Departament Analiz & i Strategii Ministerstwa Zdrowia. Mapa potrzeb zdrowotnych w zakresie kardiologii dla Polski [Health needs map for cardiology in Poland]. (2019). https://mpz.mz.gov.pl/wp-content/uploads/2019/06/MPZ_kardiologia_Polska.pdf

[122] Jaworski, R. et al. Candidemia in children after complex congenital heart defects surgery treated with caspofungin–our own experience and a review of literature. *Med. Sci. Monit. Int. Med. J. Exp. Clin. Res.* **17**, PH35–H39 (2011). 10.12659/MSM.881751 PMC3539598 21525820

[123] Jaworski, R. et al. Fungal infections in children in the early postoperative period after cardiac surgery for congenital heart disease: A single-centre experience. *Interact. CardioVasc. Thorac. Surg.* **23**, 431–437 (2016). 27222000 10.1093/icvts/ivw156

[124] Hański, W. & Burska, I. Kryptokokoza Wielonarzadowa w materiale Sekcyjnym ZAP WSzS w Radomiu. [Cryptococcal multisystemic infections in autopsies carried out at the Department of Pathology of the military hospital in Radom]. *Pol. Tyg Lek*. **44**, 253–256 (1989). 2478987

[125] European Centre for Disease Prevention and Control/WHO Regional Office for Europe. HIV/AIDS Surveillance in Europe 2023–2022 Data. (2023). https://www.ecdc.europa.eu/sites/default/files/documents/HIV-AIDS_surveillance_in_Europe_2023_%28_2022_data_%29_0.pdf

[126] Sobolewska, A., Gołab, E. & Dzbeński, T. H. Serologiczna ocena występowania zakażeń *Pneumocystis**jirovecii* u dzieci z chorobami dróg oddechowych w Polsce. [Serological evaluation of the prevalence of *Pneumocystis**jirovecii* infection in children with respiratory tract infections in Poland]. *Przegląd Epidemiol.* **63**, 359–362 (2009). 19899591

[127] Gołąb, E. et al. The occurrence of* Pneumocystis jirovecii *in people from three different age groups of Warsaw (Poland) community. *Acta Parasitol.* **53**, 106–109 (2008).

[128] Sokulska, M. et al. Genotyping of *Pneumocystis jirovecii *in colonized patients with various pulmonary diseases. *Med. Mycol.* **56**, 809–815 (2017). 10.1093/mmy/myx121 29228377

[129] Konduracka, E. Opportunistic fungal infections in patients treated with heart transplantation–own centre experiences. *Ann. Transpl.* **3**, 21–24 (1998). 10234431

[130] Jeong, W. et al. The epidemiology and clinical manifestations of mucormycosis: A systematic review and meta-analysis of case reports. *Clin. Microbiol. Infect.* **25**, 26–34 (2019). 30036666 10.1016/j.cmi.2018.07.011

[131] Kubisiak-RzepczykL, H. et al. *Scedosporium prolificans* fungaemia in a patient with acute lymphoblastic leukaemia. *J Mycol. Medicale* **23**, 261-264 (2013). 10.1016/j.mycmed.2013.08.003 24135648

[132] Samborska, M. et al. Fusarium oxysporum disseminated infection in a teenage patient with a relapse of acute lymphoblastic leukemia—case report and review of the literature. *J. Infect. Chemother.* **30**, 258-262 (2023). 10.1016/j.jiac.2023.10.011 37913869

[133] Styczynski, T. et al. Infection with *Saprochaete**clavata* in children after hematopoietic cell transplantation. *J. Pediatr. Hematol. Oncol.* **45**, e976–e979 (2023). 37278583 10.1097/MPH.0000000000002686

[134] Najwyższa Izba Kontroli. Informacja o Wynikach Kontroli. [Information on the Results of the Audit]. (2018). https://www.nik.gov.pl/kontrole/wyniki-kontroli-nik/pobierz,kzd~p_17_060_201709211008411505988521~01,typ,kk.pdf

[135] Didkowska, J. A. et al. Cancer in Poland. (2021). https://onkologia.org.pl/sites/default/files/publications/2024-01/0_krn-2023-book-2024-01-22.pdf (2023).

