# Supplementary material for: Estimated burden of serious mycoses in Poland
Source: Sci Rep. 2025 Oct 14;15:35830. doi: 10.1038/s41598-025-19690-4 (PMC12521467; doi:10.1038/s41598-025-19690-4)
Supplement: Supplementary file 1 [file 41598_2025_19690_MOESM1_ESM.odt]

Supplementary material for article:

Estimated burden of serious mycoses in Poland

Paweł M. Krzyściak1, Magdalena W. Skóra2, Agnieszka Gniadek3, David W. Denning4

# Calculation for recurrent Vulvovaginal Candidosis (rVVC) occurrence

## Vulvovaginal Candidosis (VVC) estimation

Table S1: Estimated VVC cases in Polish women based on international data

|  | Polish women population 16- 65 | Estimated all cases | Annual incidence (/50) |
| --- | --- | --- | --- |
| France 29% | 12260683 | 3555598 | 71112 |
| Italy 49% | 12260683 | 6007735 | 120155 |

The estimated cases of vulvovaginal candidiasis (VVC) among Polish women aged 16–65 using data from France and Italy and lifetime = 50 years. Foxman et. al.1

Table S2: Estimated VVC cases in Polish women based on international data

| Age group | VVC occurrence in category (% as decimal) | No of female in age group in Poland in 2023 | All estimated cases | Annual incidence (/2 y of study) |
| --- | --- | --- | --- | --- |
| 18 – 25 | 0,07 | 1409517 | 100076 | 50038 |
| 26 – 30 | 0,07 | 1070727 | 72809 | 36405 |
| 31 – 35 | 0,07 | 1307515 | 90219 | 45109 |
| 36 – 40 | 0,06 | 1531323 | 96473 | 48237 |
| 41 – 50 | 0,05 | 2969548 | 148477 | 74239 |
| 51 – 60 | 0,04 | 2312778 | 80947 | 40474 |
| Total |  | 10601408 | 589002 | 294501 |

The estimated cases of vulvovaginal candidiasis (VVC) among Polish women aged 18–60 using data from Germany (reported VVC episode in last two years). Jacob et. al.2

Table S3: Annual estimated number of VVC cases

|  | Annual estimated number |
| --- | --- |
| France | 71112 |
| Italy | 120155 |
| Germany | 294501 |
| Mean | 161,92 |

Mean annual cases of VVC in Poland based of calculation above.

## rVVC calculation

Data of rVVC occurrence:

Foxman et. al1. rVVC = 27.7% VVC

Nawrot et. al.3 rVVC = 24.7% VVC 

Denning et. al4 rVVC = 5 – 8% female population 15-50 y.

Table S4

| Source of estimation | Annual population of VVC | rVVC occurrence | Annual RVVC estimation | Mean |
| --- | --- | --- | --- | --- |
| France | 71112 | 24.7% | 17565 |  |
| France | 71112 | 27.7% | 19698 | 18631.5 |
| Italy | 120155 | 24.7% | 29678 |  |
| Italy | 120155 | 27.7% | 33283 | 31480.5 |
| Germany | 294501 | 24.7% | 72742 |  |
| Germany | 294501 | 27.7% | 81577 | 77159.5 |
| Denning* | 246474 | 0,05 | 12324 |  |
| Denning* | 246474 | 0,08 | 19718 | 16021 |
| Mean | 183060,5 |  | 35823 |  |

*  8873074 females / 36 years life time

# Estimation Candida Bloodstream Infection (BSI) cases in Poland

Estimated BSI cases using various methods, such as single-centre data, patient-days, and ICU approaches.

Recalculation from patients/day factor to number of cases – used formulas

### Data:

Annual Case (C): ?

Turnover Rate (W): 44.8 patients per bed per year.

Number of Beds (L): 164,400.

Average Length of Stay (S): 5.1 days.

Case Rate (P): 0.04 +/- 0.03 cases per 1,000 patient-days.

### Simply method:

formuła

Maximum Patient-Days = L ×365 = 164,400×365 = 60,006,000 

### C = 0.00004×60,006,000 = 2,400.24 

### This method is simpler but less accurate because it assumes the maximum utilization of beds in the hospital, which is rarely achievable in real-world conditions.

### 

### Formula for Annual Cases:

C = 0.00004 × 44.8 × 164,400 × 5.1 = 1,502 

  - 

Table S5: Table of Estimated Cases of Candida BSI in Poland

| Estimation Method | Lower Range | Mean Value | Upper Range |
| --- | --- | --- | --- |
| Based single centre (0.061%) | - | 4,210 | - |
| Based on patient-days (patient numbers/average stay) base 0.04 per 1000 pts/day | 600 | 2,400 | 4,200 |
| Based on patient-days (bed numbers/turnover rate) Base 0.04 per 1000 pts/day | 380 | 1,500 | 2,630 |
| Based on ICU (direct approach) | 280 | 3,180 | 4,590 |
| Based on ICU (indirect approach) | 1,760 | 1,830 | 2,500 |
| Mean | 755 | 2624 | 3480 |

Explanations:

    1. Based on patient numbers (0.061%):

        ◦ Estimate based on the total number of hospitalized patients (6,895,900) and an incidence rate of 0.061%.

        ◦ Result: 4,210 cases (no range available).

      3. Based on ICU:

        ◦ Direct approach: Based on Candidaemia incidence in ICU (0.08%–1.3%), extrapolated to all hospitals assuming ICU accounts for 30.8% of cases:

            ▪ Range: 280–4,590 cases.

            ▪ Mean: 3,180 cases.

        ◦ Indirect approach: Accounts for overall BSI incidence in ICU and Candida’s share in BSI:

            ▪ Range: 1,760–2,500 cases.

            ▪ Mean: 1,830 cases.

# Estimation of Esophaegal Candidosis (EC) – overlaping population

Using the inclusion-exclusion principle:

T= |H| + |G| + |L| − |H∩G| − |H∩L| − |G∩L| + |H∩G∩L|

Data and Definitions

Variables:

- T = Total number of patients with Esophageal Candidosis EC

- H = Number of patients with Human Immunodeficiency Virus and EC (HIV+EC)

- G = Number of patients with GastroEsophageal Reflux Disease and EC (GERD+EC)

- L = Number of patients with Liver Cirrhosis and EC (LC+EC)

Ranges:

- H: 347 ≤ H ≤ 1462

- G: 4700 ≤ G ≤ 12500

- L: 69 ≤ L ≤ 501

Overlap (shared groups):

-  H∩G=0.8H

-  H∩L=0.3H

-  L∩G=0.37L

Triple overlap:

-  H∩G∩L=x, to be calculated.

### Logical Constraints

- Non-Negativity of Sets:

  - x≥0

- Subset Limitations:

  - x≤∣H∩G∣

  - x≤∣H∩L∣

  - x≤∣G∩L∣

- Constraint from ∣H∪G∪L∣:

  - ∣H∪G∪L∣≤H+G+L: The total number of elements in ∣H∪G∪L∣—without accounting for repeated intersections—cannot exceed the sum of the sizes of H, G, and L.

#### Minimum Value x:

The minimum x is determined by the relationship between H, G, L, and ∣H∪G∪L∣—without the intersection of all three sets:

xmin​=max(0,H+G+L−∣H∪G∪L∣without x​)

Where:

∣H∪G∪L∣without x​=∣H∣+∣G∣+∣L∣−∣H∩G∣−∣H∩L∣−∣G∩L∣

The maximum x is constrained by the smallest value among the subsets and the logical limit of ∣H∪G∪L∣: 

xmax​=min(∣H∩G∣,∣H∩L∣,∣G∩L∣,H+G+L−∣H∪G∪L∣without x​)

### Calculation Procedure

- Calculate ∣H∪G∪L∣without x​:

- ∣H∪G∪L∣without x​=∣H∣+∣G∣+∣L∣−∣H∩G∣−∣H∩L∣−∣G∩L∣

- Determine Minimum x:

- xmin​=max(0,H+G+L−∣H∪G∪L∣without x​)

- Determine Maximum x:

- xmax​=min(∣H∩G∣,∣H∩L∣,∣G∩L∣,∣H∣+∣G∣+∣L∣ - H∪G∪L∣without x​)

Table S6: Overlap Among EC Patients Subgroups (HIV+EC, GERD+EC, LC+EC)

|  | Min | Average | Max |
| --- | --- | --- | --- |
| H | 347 | 905 | 1462 |
| G | 4700 | 8600 | 12500 |
| L | 69 | 285 | 501 |
| H ∩ G | 278 | 724 | 1170 |
| H ∩ L | 104 | 271 | 439 |
| L ∩ G | 26 | 105 | 185 |
| H ∩ G ∩ L | 0 | 93 | 185 |
| H ∪ G ∪ L | 4708 | 9054 | 12695 |

Figure 1 The Venn diagram illustrates the overlaps for min, mean and max number of cases. Each circle represents one group (HIV+EC, GERD+EC, LC+EC), and the overlaps indicate the shared populations, including the triple intersection. source: figure generated by RStudio package eulerr (R)

# Estimation of Invasive Pulmonary Aspergillosis (IPA) cases

IPA among transplant patients estimation

Table S7: IPA among transplant patients estimation

| Transplant | IPA Incidence | No pts in Poland | Estimation in Poland |
| --- | --- | --- | --- |
|  | min | max |  | min | max | mean |
| SOT | Neofytos 20215 | PT 20196 |  |  |  |
| Renal | 0,01 | 0,04 | 977 | 12 | 39 | 25 |
| Liver | 0,01 | 0,05 | 523 | 5 | 25 | 15 |
| Heart | 0,04 | 0,27 | 179 | 6 | 48 | 27 |
| Lungs | 0,08 | 0,23 | 99 | 8 | 23 | 16 |
| Total |  |  | 1778 | 31 | 135 | 83 |
| alloHSCT | Data from Poland |  |  |  |  |
| Adult | 0,08 | 0,16 | 706 | 59 | 111 | 85 |
| Pediatric | 0,02 | 0,02 | 84 | 1 | 2 | 1 |
| Total |  |  | 790 | 60 | 112 | 86 |

alloHSCT – allogeneic Hematopoietic Stem Cell Transplantation; IPA  - Invasive Pulmunary Aspergillosis; pts – ptients; PT 2019 – Data from Poland – Poltranplant; SOT - solid organ transplantation

Invasive Pulmonary Aspergillosis (IPA) among Interstitial Lung Disease (ILD)

Table S8: IPA among ILD calculation adjusted data from Liu et al.7 with NFZ rapport 8

| Liu's ILD category | ICD10 Liu's adjusted to NFZ rap. | Liu base | NFZ PL estimation |  |
| --- | --- | --- | --- | --- |
| Total | IPA | %IPA | No ILD pts | cor** | Cor No ILD pts | Est. No IPA |
| IPF | J84.1 w/o  M | 118 | 21 | 45886 | 6359* |  | 6359 | 1132 |
| CTD-ILD |  | 227 | 37 | 45732 | 5078 |  | 5188 | 1451 |
|  | IPAF | M32 lupus er. + M35 other than M35.0 | 80 | 13 | 45732 | 1539 | 27,5 | 1594 | 259 |
|  | RA-ILD | M05 | 26 | 11 | 42.3 | 2222 | 25 | 2247 | 951 |
|  | pSS-ILD | M35.0 | 61 | 4 | 45814 | 231 | 0 | 231 | 15 |
|  | ANCA-ILD | I77.82 | 11 | 5 | 45.5 | nd |  | 0 | 0 |
|  | IIM-ILD | M33 | 45 | 3 | 45844 | 275 | 15 | 290 | 19 |
|  | Ssc-ILD | M34 | 4 | 1 | 25.0 | 811 | 15 | 826 | 207 |
|  | COP |  | 7 | 0 | 0.0 | ND | ND | 0 | ND |
| Total | 352 | 58 |  | 11436,5 | 110 | 16734,5 | 2582 |

IPF - Idiopathic pulmonary fibrosis; CTD-ILD Connective tissue disorders

IPAF interstitial pneumonia with autoimmune features; RA-ILD Rheumatoid arthritis; pSS-ILD primary Sjögren′s syndrome; ANCA-ILD  antineutrophil cytoplasmic antibody (ANCA)–associated vasculitis; IIM-ILD  idiopathic inflammatory myopathy /dermatopolymyositis; Ssc-ILD systemic sclerosis, COP - not expleined in Liu's work - chronic obstructive pneumoniae?

**correction =  number records with  „<5” cases × 2.5

1. 	Foxman B, Muraglia R, Dietz JP, Sobel JD, Wagner J. Prevalence of recurrent vulvovaginal candidiasis in 5 European countries and the United States: results from an internet panel survey. J Low Genit Tract Dis. 2013;17(3):340-345.

2. 	Jacob L, John M, Kalder M, Kostev K. Prevalence of vulvovaginal candidiasis in gynecological practices in Germany: A retrospective study of 954,186 patients. Curr Med Mycol. 2018;4(1).

3. 	Nawrot U, Grzybek-Hryncewicz K, Zielska U, Czarny A, Podwińska J. The study of cell-mediated immune response in recurrent vulvovaginal candidiasis. FEMS Immunol Med Microbiol. 2000;29(2):89-94.

4. 	Denning DW, Kneale M, Sobel JD, Rautemaa-Richardson R. Global burden of recurrent vulvovaginal candidiasis: a systematic review. Lancet Infect Dis. 2018;18(11):e339-e347.

5. 	Neofytos D, Garcia-Vidal C, Lamoth F, Lichtenstern C, Perrella A, Vehreschild JJ. Invasive aspergillosis in solid organ transplant patients: diagnosis, prophylaxis, treatment, and assessment of response. BMC Infect Dis. 2021;21(1):296.

6. 	POLTRANSPLANT. Biuletyn Informacyjny. Centrum Organizacyjno-Koordynacyjne ds. Transplantacji Poltransplant; 2024.

7. 	Liu Y, Jiang H, Zhao T, et al. Clinical features and risk factors of invasive pulmonary aspergillosis in interstitial lung disease patients. BMC Pulm Med. 2024;24(1):602.

8. 	Centrum e-Zdrowia. Epidemiologia choroby śródmiąższowej płuc. Published online September 3, 2021.
